# Mechanisms of DBS: from informational lesions to circuit modulation and implications in OCD

**DOI:** 10.3389/fnhum.2025.1492744

**Published:** 2025-05-08

**Authors:** Julia M. Shea, Chaim M. Feigen, Emad N. Eskandar, Nathaniel J. Killian

**Affiliations:** ^1^Albert Einstein College of Medicine, Bronx, NY, United States; ^2^Department of Neurological Surgery, Montefiore Medical Center, Bronx, NY, United States

**Keywords:** obsessive-compulsive disorder, psychosurgery, deep brain stimulation, CSTC circuitry, neuromodulation

## Abstract

In 2009, treatment-resistant obsessive compulsive disorder (OCD) was approved as an indication for deep brain stimulation (DBS) under a Humanitarian Device Exemption (HDE). This review examines the mechanisms by which DBS produces its effects, focusing on its interaction with the pathophysiology of OCD, a condition thought to involve overactive cortico-striatal-thalamo-cortical (CSTC) circuits. We first review initial theories of excitation and inhibition. We then transition to discussion of the “informational lesion” hypothesis, which suggests that DBS may prevent the transmission of normative neural activity through the stimulated region. Specifically, high-frequency stimulation may disrupt pathological network patterns by masking or antidromically blocking synaptic inputs. Another hypothesis suggests that DBS disrupts network activity by driving action potentials antidromically, which activates upstream inhibitory interneurons and imposes rhythmic activity on local regions based on DBS stimulation parameters. Recent animal studies support these theories of disruption of pathological network activity, showing that high-frequency DBS can prevent neurons from responding to intrinsic oscillations, and thereby relieve OCD symptoms. This review also discusses the variable effects of DBS, noting immediate improvements in mood and anxiety, with with a more gradual reduction in OCD symptoms. These differential findings suggest that DBS may produce its effects through both immediate neuromodulation as well as long-term synaptic remodeling. In summary, this review synthesizes the current mechanistic understanding of DBS, focusing on OCD, and highlights areas of discrepancy between studies and opportunities for future research. A deeper mechanistic understanding of DBS could lead to more optimized and effective treatment, improving outcomes for patients with treatment-refractory OCD as well as other psychiatric conditions.

## Introduction

OCD affects an estimated 2.3% of the population, with selective serotonin reuptake inhibitors (SSRIs) and cognitive behavioral therapy—specifically, exposure and response prevention (ERP)—offered as first-line treatments (Koran et al., 2007; Ruscio et al., 2010). SSRIs are of significant benefit in 40–50% of patients, and ERP is estimated to have between a 43 and 50% remission rate and between 62 and 65% response rate, (Ost et al., 2015; Pittenger, 2021). Despite these treatments, it is thought that 10% of patients remain severely impaired despite optimal psychiatric care (Denys, 2006).

Based on the positive results of anterior capsulotomy, deep brain stimulation (DBS) for treatment-refractory OCD was first attempted in 1999, leading to the publication of a seminal case report. Unlike capsulotomy, DBS is reversible and modifiable. Transcranial magnetic stimulation, which was approved for OCD in 2018 and has shown a 38.1% response rate after six weeks of stimulation and 45.2% at one month follow up, is a non-invasive alternative (Carmi et al., 2019).

In the case report, a 39-year-old woman suffering from severe OCD for more than 20 years was reported to have almost instantaneous relief from anxiety and obsessive thinking, which disappeared when stimulation was turned off (Nuttin et al., 1999). During the subsequent two weeks of constant stimulation, her parents reported that about 90% of her compulsive behavior and rituals had vanished (Nuttin et al., 1999). In 2008, a double-blind crossover study was conducted to compare sham and active stimulation of the STN, based on its efficacy in reducing repetitive behaviors, anxiety, and obsessive-compulsive symptoms in patients with Parkinson’s (Mallet et al., 2008). The landmark study found significant reductions in OCD symptoms and increases in global functioning with active but not sham stimulation. It should be noted that 15 serious adverse events were reported across the 17 patients who underwent the surgery, some related to the surgery itself such as infection and others related to stimulation such as transient hypomanic status or anxiety (Mallet et al., 2008). As a result of these and other promising findings in 2009, treatment-refractory OCD was approved via HDE as an indication for DBS targeting the ventral anterior limb of the internal capsule (ALIC) (Greenberg et al., 2010; Haber et al., 2021; Pinckard-Dover et al., 2021). A 2015 meta-analysis of studies of DBS for OCD that included the results of DBS of various stimulation sites found a 60% response rate overall (Alonso et al., 2015).

Despite this positive result, the underlying mechanisms driving the efficacy of DBS in OCD remain under investigation. In contrast to movement disorders, OCD is among the least studied indications for DBS, with few animal studies devoted to the application of the technology to the disorder (Zhang et al., 2024). However, recent studies lend support to the theory that DBS disrupts pathological network activity in OCD (Lowet et al., 2022; van den Boom et al., 2023).

This review will first outline the pathophysiology of OCD and the neurobiological markers of its successful treatment. Following this overview, we will review the early 2000s history of how researchers arrived at the prevailing “informational lesion” theory of DBS and discuss how it might be relevant to OCD. Finally, we will explore the immediate and long-term effects of DBS for OCD.

## Pathophysiology of OCD: CSTC circuits

Clinical understanding of OCD suggests that the disorder involves excessive anticipation of negative outcomes, leading to compulsive actions to prevent these negative outcomes (Fradkin et al., 2020). Prefrontal cortical networks are proposed to contribute to this model of OCD, as the lateral orbitofrontal cortex (OFC) processes negative reinforcers and fear response and the OFC more broadly is associated with anticipating outcomes and generating related goal-directed behavior (Kringelbach and Rolls, 2004). Indeed, prefrontal hyperactivity, especially in the OFC, is linked to OCD symptomatology (Ahmari and Rauch, 2022), and the degree of overconnectivity between the PFC and its striatal target has been shown to predict symptom severity (Graybiel and Rauch, 2000; Milad and Rauch, 2012; Simon et al., 2010; Chen et al., 2022). Notably, it has been hypothesized that baseline prefrontal cortex hyperactivity may interfere with the recruitment of this area during tasks that require its participation, perhaps explaining the deficits observed in patients with OCD on various cognitive tasks (Ahmari and Rauch, 2022).

This prefrontal cortex overactivity may be related to dysfunction of cortico-striatal-thalamo-cortical (CSTC) circuits, a network involving the anterior cingulate cortex (ACC), orbitofrontal cortex (OFC), dorsolateral prefrontal cortex (dlPFC), ventral striatum, mediodorsal thalamus (MD), and amygdala (Bourne et al., 2012). These circuits, which project from frontal-cortical regions to the striatum, then to thalamic sites, and finally back to the cortex (Milad and Rauch, 2012), are proposed as central to the pathophysiology of OCD (Pauls et al., 2014; Alexander et al., 1986). CSTC circuits are classified as either direct or indirect depending on their net effect on the thalamus, resulting in either increased (i.e., direct pathway) or decreased cortical excitation (i.e., indirect pathway). An imbalance between direct and indirect pathways is thought to contribute to OCD, with overactivity of the direct pathway creating a positive feedback loop perpetuating CSTC circuit hyperactivity.

Four distinct CSTC circuits have been hypothesized to be involved in OCD, (1) “the affective circuit” which is involved in emotion and reward-associated processing, (2) the “dorsal cognitive circuit” which is related to executive function including working memory, (3) the “ventral cognitive circuit” which is responsible for motor and response inhibition, and (4) the “sensorimotor circuit” which has been proposed to be involved in habit-based behavior contributing to compulsivity (Fineberg et al., 2018; Milad and Rauch, 2012; Van Den Heuvel et al., 2005). Evidence has accumulated suggesting that targeting specific circuits may lead to varying clinical effects (Tyagi et al., 2019).

Studies of DBS in rats exposed to mild chronic stress have shown that targeting different brain regions improves different aspects of mood-related behaviors (Lim et al., 2015). Stimulation of particular brain areas has been shown to enhance motivational aspects of behavior and reduce anxiety levels, while stimulation of another region has been shown to enhance hedonia and reduce behavioral despair. Although these studies were focused on depression, the differences in clinical outcome depending on brain target suggest that the choice of target for DBS should depend on the key symptoms to be treated rather than an intention to resolve a complex and multifaceted disorder (Lim et al., 2015). These four distinct circuits may represent different anatomical targets in OCD.

**Figure 1:**
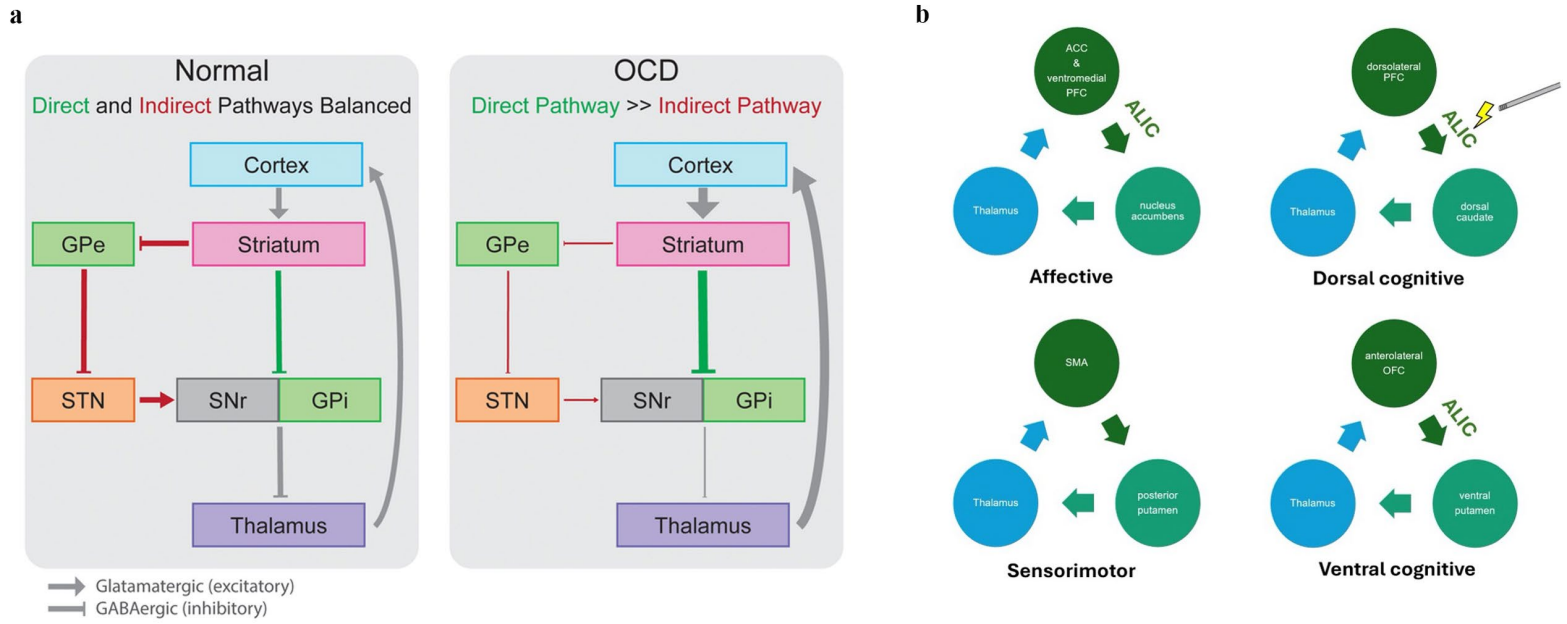
Circuits involved in OCD pathophysiology. **(a)** In the normally functioning cortico-striato-thalamo-cortical circuit, direct (green) and indirect (red) pathways lead to increased or decreased inhibition of the thalamus, respectively, in a balanced manner. In OCD, the direct pathway is overactivated relative to the indirect pathway, leading to pathological overactivity of cortical regions through an excitatory loop. GPe, globus pallidus externa; GPi, globus pallidus interna; SMA, supplementary motor area; SNr, substantia nigra pars reticulata. Adapted from Karas et al. (2018), Front Neurosci with permission. **(b)** The figure depicts four distinct CSTC circuits: (1) the affective circuit involved in emotion and reward processing, (2) the dorsal cognitive circuit related to executive function and working memory, (3) the ventral cognitive circuit responsible for motor and response inhibition, and (4) the sensorimotor circuit associated with habit-based behavior contributing to compulsivity. Stimulation of a subsection of the ALIC that passes by the ventral striatum is associated with clinical improvement in OCD, potentially because of its ability to modulate these circuits (Baldermann et al., 2019). The medial dorsal nucleus of the thalamus and the anterior STN both connect to this tract, which may explain the efficacy of multiple stimulation sites in DBS for OCD. Though the ALIC does not connect the SMA and posterior putamen, stimulation of the ALIC may affect the posterior putamen, thereby indirectly modulating this circuit.

Understanding the pathophysiology of OCD provides a framework for interpreting the neurobiological outcomes of DBS treatment for this disorder. Across neuroimaging studies, it has been shown that there is a normalization of hyperactivity in the OFC, ACC, and mPFC after effective exposure therapy, pharmacotherapy, or DBS (Ahmari and Rauch, 2022). These cortical changes represent important clinical markers but are likely the reflection of more subterranean alterations in circuitry. Indeed, the extent to which DBS reduces the overconnectivity between the PFC and its striatal targets appears to correlate with the degree of symptom relief (Figee et al., 2013) (see Figure 1).

### Stimulation targets of DBS for OCD

The initial target of DBS for OCD was the ALIC. The adjacent junction of the ventral capsule and ventral striatum (VC/VS) followed soon afterward as a target based on the surgical target of capsulotomy (Greenberg et al., 2010). Similar to the evolution of capsulotomy surgery for OCD, the VC/VS stimulation site has become more posterior over time (Greenberg et al., 2010). Fibers within CSTC circuits become denser as they course posteriorly toward the thalamus, allowing for more clinical effect with the same degree of stimulation (Kopell et al., 2004; Velasco et al., 2006). Additional data suggested that an even more posterior and inferior target such as the caudal nucleus accumbens (NAc) could also be effective as a target for OCD (Sturm et al., 2003; Van Kuyck et al., 2003). The subthalamic nucleus (STN) and the inferior thalamic peduncle are also common targets (Tierney et al., 2014). Recent attention has turned to the bed nucleus of the stria terminalis (BNST) as a potentially more effective site than the ALIC because stimulation of the BNST may affect a wider subset of white matter tracts in the ventral capsule and thereby engage a more diffuse cortical/subcortical network (Karas et al., 2018). In addition, the BNST is thought to play a role in fear conditioning (Goode et al., 2019).

In both ALIC and STN DBS, it has been shown that the stimulation of a tract connecting the dorsal anterior cingulate (dACC) and ventrolateral prefrontal cortices with the anteromedial STN is predictive of clinical outcome (Li et al., 2020). The dACC is thought to be the origin of the limbic hyperdirect pathway, which directly connects the cortex to the STN, bypassing the striatum. Animal studies have shown that lesions to the limbic hyperdirect pathway result in diminished discriminative accuracy and increased perseveration—behaviors relevant to OCD. In contrast, direct lesions to the dACC itself in humans, as performed in anterior cingulotomy procedures, lead to improvement in OCD symptoms (Dougherty et al., 2002). These findings suggest that the activity of the hyperdirect is causally related to OCD-like behaviors, and directly reducing pathological hyperactivity within the dACC can have therapeutic benefits.

Typically, DBS parameters are selected by using computational models that take into account information about target volume, target elements, and parameters that have been safe in the past (Lozano et al., 2019; van Westen et al., 2021). Visible emotional response is also often used as a measure of effectiveness (Widge and Dougherty, 2022). Most trials of DBS for OCD have used “open loop” DBS in which stimulation is delivered continuously without biofeedback. The setting of parameters often includes an initial period trial and error, with one case report discussing holding frequency constant at 135 Hz and varying pulse width and amplitude to avoid unfavorable side effects such as feelings of clamminess or nausea (Beydler et al., 2023). However, despite technology that can visualize the region of potential tissue activation, it remains difficult to verify target engagement, a term first used in drug development, that refers whether the given intervention interacted with and changed the biological substrate it was designed to change.

The VC/VS target used in OCD is made up of nuclei and a white matter tract containing fibers from almost the entire prefrontal cortex. Furthermore, the anatomy is heterogeneous across patients. Even if anatomy is standardized, with four controllable parameters, arbitrary combinations of 4–8 electrodes within the anatomic target, and no objective feedback, it is difficult to prove target engagement. Furthermore, current methods of assessing target engagement, such as assessing for visible emotional response, may have little validity, as there is no evidence that emotional response is required for or predictive of clinical outcome (Widge et al., 2018; Widge and Dougherty, 2022).

In contrast, closed loop technologies sense electrical activity, identify markers of a particular state, and automatically deliver or adjust stimulation to alter that electrical state. For instance, if the neurobiological signature of lapses in cognitive control are sensed, closed-loop DBS can intervene (Widge, 2023). Although most studies of DBS for OCD have used open loop technology, closed loop may be able to augment treatment response. Adaptive DBS takes closed loop DBS one step further and incorporates not only “central” biomarkers such real time brain activity but also “peripheral” biomarkers, such as tremor in the case of Parkinson’s.

There has also been recent innovation in optimizing site targeting. fMRI tractography has allowed for more precise visualization of individual anatomy and targeting of electrodes to specific sites associated with positive response (Middlebrooks et al., 2020). Biomarkers such as evoked potentials in response to stimulation have also been found to have predictive value for treatment response and can help guide site targeting (Peeters et al., 2022). In summary, various recent advancements in the field of DBS have allowed for improved target engagement and target selection.

Despite these advancements, not all patients with OCD respond to DBS. While specific differences in anatomy between non-responders, poor responders, and responders have not been studied to date, the reasons that some patients respond while others do not may have to do with the composition of the brain region stimulated. As will be discussed, because DBS is thought to cause release of neurotransmitters at the site of stimulation, excite local axons, and propagate antidromic action potentials, the effects of DBS may be dependent on the nature of the synapses in and connectivity of the stimulated nucleus. Theoretically, a stimulated nucleus that is highly connected to other structures involved in CSTC loops, for example, may allow for a more effective “block” in the transmission of information underlying the OCD phenotype.

### The inhibition hypothesis

We now transition to a discussion of how DBS may work mechanistically. Given basic physiological principles, one might expect that electrical stimulation of axons and cell bodies near the tip of an electrode would result in increased firing of the axons projecting away from the region stimulated (Dostrovsky and Lozano, 2002). However, because DBS has been observed to affect patients in a manner similar to a pharmacological lesion such as by microinjection of lidocaine, or surgical removal of brain tissue, it was initially thought that DBS causes an inhibition of local neural activity (Lowet et al., 2022; Dostrovsky and Lozano, 2002).

Ultimately, the scientific community would converge on a theory of “informational lesions” lesions that reconciles the appearance of inhibition with other data that supported excitation, but early animal experiments supported the hypothesis that DBS reduces neural activity. Animal studies showed that high-frequency stimulation of the STN, globus pallidus internus (GPi), and thalamus resulted in dampening of neuronal firing in the targeted region (Bourne et al., 2012). Single-unit recordings in studies of STN and GPi DBS in humans also showed local inhibition (Bourne et al., 2012).

A few theories have been proposed to describe how stimulation could lead to neural depression. Proposed mechanisms include depolarization-mediated blockade of voltage-gated currents (Beurrier et al., 2001) and GABA release from afferent synaptic terminals (Lafreniere-Roula et al., 2010). The depolarization blockade theory suggests that high-frequency stimulation may cause sustained depolarization of neural membranes, preventing the initiation or propagation of action potentials. The GABA release hypothesis suggests that stimulation may activate afferent inhibitory terminals on the cell body. This hypothesis was supported by the finding that stimulation of regions with primarily excitatory afferents were activated instead of inhibited (Dostrovsky and Lozano, 2002).

However, the depolarization blockade theory was incompatible with the relatively long recovery time of stimulated regions, and the observation that firing rates were reduced but not blocked during GPi stimulation (Dostrovsky et al., 2000; Dostrovsky and Lozano, 2002). The reduced rather than silenced firing suggests that there may be a more graded mechanism of inhibition, such as GABA release, which may also involve a longer recovery time. The GABA hypothesis was weakened by the finding that when the globus pallidus externa (GPe), whose inhibitory input to the STN was thought to be causing depression of the downstream SNr and GPi, was lesioned out, the downstream depression continued (Benazzouz et al., 2000; Benazzouz et al., 1995). Thus, there may be direct inhibition of the soma, whether by depolarization blockade or another mechanism. In sum, to the extent that DBS causes inhibition, it is likely to be doing so through a combination of mechanisms such that these various results can be seen.

### The excitation hypothesis

Other studies suggested that DBS was excitatory rather than inhibitory. Seminal animal studies of DBS showed that stimulation of the rat STN produced an increased discharge rate of some STN neurons (Maurice et al., 2003), which was thought to be caused by the activation of glutamatergic afferents. Recordings during high-frequency stimulation of the STN rats and monkeys have also shown increased output to nuclei from the targeted region, through either orthodromic or antidromic action potentials (Hashimoto et al., 2003; Maurice et al., 2003; Windels et al., 2000).

One explanation for the neuronal excitation is proposed by a computational approach using a cable-based neuronal model. This model shows that within 1.5 mm of the electrode center, direct stimulation of the neuron would trigger efferent axonal output while the cell body showed suppression of activity (McIntyre et al., 2004a). However, at 2 mm, the stimulation would not be sufficient for efferent axonal output, and stimulation-induced presynaptic output on the neuron would lead to its suppression. This is thought to be because the threshold for afferent inputs projecting to the region of the electrode is lower than the threshold for direct activation of local cells (Baldissera et al., 1972; Dostrovsky et al., 2000; Gustafsson and Jankowska, 1976; Jankowska et al., 1975; McIntyre and Grill, 2002). Summation of an overall inhibitory synaptic effect on the cell body can suppress somatic firing (Dostrovsky et al., 2000). However, because action potential initiation begins in the axon, efferent output of neurons supra-threshold for direct activation is unaffected by the trans-synaptic inhibition (Nowak and Bullier, 1998b; Nowak and Bullier, 1998a; McIntyre and Grill, 1999; McIntyre et al., 2004b).

Separately, depending on a neuron’s orientation and position relative to the electrode, the soma can be directly hyperpolarized by the stimulus pulse (McIntyre et al., 2004b). Computational models support the idea that DBS directly stimulates axons but if this stimulation is sub-threshold, neurons can exhibit suppression of their intrinsic firing patterns regulated by either stimulation-induced trans-synaptic inputs, or direct hyperpolarization of the soma. Thus, some of the inhibition observed in early research may be related to recording cell bodies receiving inhibitory synaptic input, or recording regions downstream of axons that are sub-threshold such that the state of the cell body does determine the firing pattern of the axon.

At this point there was a paradox. The discussion had come full circle: How could axonal excitation cause a clinical effect resembling ablation? One possibility was that the stimulated neurons cannot maintain high-frequency action due to neurotransmitter depletion (Urbano et al., 2002). However, several *in vivo* experiments demonstrated sustained changes in neuronal firing (Windels et al., 2000; Hashimoto et al., 2003; Anderson et al., 2003; Windels et al., 2003). Attention turned instead toward the hypothesis that DBS results in modulation of pathological network activity.

### A revised theory: the informational lesion

The “informational lesion” theory of DBS addressed the paradox of how excitation could resemble ablation. It was proposed that DBS may, through stimulation, interfere with the ability of neurons to respond to synaptic inputs, thereby creating an informational lesion that disrupts pathological network patterns and produces a clinical result that resembles ablation (Grill et al., 2004). Neurons can follow certain rhythmic inputs, a phenomenon known as entrainment, and an important network communication mechanism (Fries, 2015; Buzsaki and Draguhn, 2004). Entrainment by DBS has been proposed as a potential therapeutic mechanism, whereby DBS-mediated entrainment interferes with the ability of neurons to process synaptic inputs and follow intrinsic brain rhythms (Grill et al., 2004).

In a seminal 2004 paper proposing the informational lesion hypothesis of DBS, Grill et al. discussed that the frequency dependence of DBS is the result of the interaction between DBS and the intrinsic neuronal activity of the stimulated area (Grill et al., 2004). Low-frequency stimulation evoked activity that approximately superposed with the intrinsic activity of the neuron, whereas high-frequency stimulation allowed for the intrinsic activity to be effectively masked by the stimulus train. The higher the intrinsic firing rate of the neuron, the higher the DBS frequency needed to be for this masking effect to take place.

A recent review article suggests that DBS may be a generic tool to override low frequency stimulation, such as those underlying tremor and akinesia-rigidity (Lozano et al., 2019). Axon terminals may see a depletion of neurotransmitters, but even if this does not occur, information processing theories suggest that the synapses will become low-pass filters, whereby the transmission of low-frequency activity is suppressed (Lindner et al., 2009; Llinas et al., 2002; Rosenbaum et al., 2014). Like informational lesions, low-pass filtering is an extension of the excitation hypothesis of DBS.

### Emerging evidence from animal studies: high-frequency DBS prevents propagation of oscillations

Lowet et al. (2022) contributed a study supporting the informational lesion hypothesis by studying the effects of DBS on individual hippocampal cells in awake mice. Although the hippocampus is not the target of DBS for OCD, the study remains relevant for understanding how DBS works mechanistically. Lowet et al. delivered either high-frequency, clinically effective 140 Hz DBS or the less effective 40 Hz DBS for 1 s to awake mice. They found that 40 Hz DBS powerfully entrained neuronal membrane voltage and spike rate to the 40 Hz frequency, and 140 Hz DBS did similarly but to a lesser extent. They attributed this finding to the biophysical limitations of neurons: 40 Hz stimulation allows neurons to be affected by subsequent DBS electrical pulses in a more predictable fashion than 140 Hz stimulation, in which a neuron is still affected by previous pulse when a new one is incoming.

In the hippocampus, there are prominent and persistent theta frequency (3–12 Hz) oscillations that are crucial for hippocampal-dependent spatial memory and spatial navigation. Using optogenetics to mimic these oscillations, Lowet et al. showed that DBS prevented neurons from entraining, as would usually be expected (McIntyre and Anderson, 2016). The researchers proposed that stimulation-induced action potentials may outnumber intrinsically generated action potentials leading to a masking effect, or stimulation-induced action potentials traveling antidromically may collision block intrinsically generated action potentials traveling orthodromically (McIntyre et al., 2004a).

According to an original paper outlining the informational lesion hypothesis, since stimulation frequency remains constant during DBS (i.e., 100 Hz), the informational content of the stimulation signal is effectively zero, generating an informational lesion in the circuit (Grill et al., 2004). However, according to Lowet et al., the frequency of neurons stimulated by high-frequency 140 Hz DBS is not simply 140 Hz given the biophysical limitations of neurons. Nevertheless, the electrical coherence may be as content-free as the informational content encoded by a constant frequency. It may be that it is not entrainment to a uniform high frequency that is necessary to create an informational lesion but the effect of high-frequency DBS on blocking incoming action potentials or masking intrinsic activity. Lowet et al. further argued that having neurons entrained by DBS to a specific rhythm, as in the case of the 40 Hz DBS, may cause network over-synchronization that produces unwanted side effects. A less “electrically coherent” pattern result may allow for a purer informational lesion.

### Emerging evidence from animal studies: excitation-based informational lesions

Another recent, study utilizing SAPAP3^−/−^ mice, a well-established animal model for OCD, also offers support for an informational lesion based in excitation (van den Boom et al., 2023). The SAPAP3^−/−^ animal model exhibits compulsive-like grooming which abates in a dose-dependent manner with DBS of the mouse internal capsule.

Looking under the hood to determine the cause of this eventual suppression and reduced grooming activity, the study found that a subset of neurons in the prefrontal cortex and their striatal targets exhibited transient excitation or inhibition immediately upon DBS onset, and another subset exhibited excitation or inhibition. The researchers hypothesized that the sustainedly recruited neurons were responsible for the reduction in grooming, as a correlation was observed between the number of sustainedly recruited neurons in the mOFC and lOFC and the degree of grooming reduction.

The researchers identified neurons that were modulated specifically during grooming in SAPAP3^−/−^ mice. The number of neurons in the mOFC active during grooming was consistently reduced by DBS across DBS-parameter experiments. Thus, in what seems to be a paradox, by primarily exciting neurons in the mOFC, neurons typically involved in mediating the grooming behavior may have been taken out of a circuit. Using optogenetic techniques on these mOFC grooming-associated neurons, the researchers mimicked DBS and again observed a reduction in grooming behavior, suggesting that functionally knocking out these neurons played a causal role in behavior change. With mOFC neurons unable to respond to intrinsic synaptic input, as suggested by the informational lesion hypothesis, circuits associated with grooming may be undercut, reducing the strength of grooming circuits and overall cortical activity despite excitation of a specific population of neurons (van den Boom et al., 2023).

It should be noted that some research has suggested that afferent and efferent synapses cannot maintain the initial level of activation that DBS produces (von Gersdorff and Borst, 2002). That is, although DBS may cause short-term activation, there is eventual suppression of activity due to synaptic transmission failure. This idea suggests that DBS may create a sort of informational lesion, but one that is based in transmission failure at the output sites. This synaptic failure concept harkens back to the original inhibition hypothesis but focuses on the effects at output regions rather than on the cells directly stimulated by DBS.

### An alternative network-based explanation: antidromic activation of inhibitory interneurons to restore circuits

Another hypothesis as to how DBS may transform circuitry to a nonpathological state is through increased drive onto inhibitory interneurons. A rat study on DBS of the NAc – a common site of electrode placement for OCD – showed a potentiation of the response of interneurons to subsequent stimulation of the NAc and MD (McCracken and Grace, 2007). Interneurons may be stimulated by the driving of corticostriatal antidromic action potentials, and high-frequency stimulation may be capable of inducing lasting changes in the behavior of these interneurons through a long-term potentiation-like mechanism (McCracken and Grace, 2007).

A follow-up study by the same authors showed that NAc DBS delivered for 90 minutes increased spontaneous and induced fast (beta and gamma) coherence within and between the mPFC, lOFC, MD and NAc, regions comprising a circuit that often exhibits aberrant metabolism in patients with OCD (McCracken and Grace, 2009). On a cellular level, fast rhythmic activity is generated via synchronous inhibition within networks of inhibitory interneurons, with frequency and duration modulated by glutamatergic input. The authors propose that increased coherence produced by high-frequency DBS may be due to LTP-mediated increased drive onto interneurons, synchronizing inhibition across cortical and subcortical networks, thereby normalizing function of a circuit that shows aberrant activity in OCD. In short, the authors provide an alternative explanation for the alterations in network activity based on the concept of DBS directly inducing synaptic changes in intrinsic circuitry, rather than masking intrinsic circuity.

**Figure 2:**
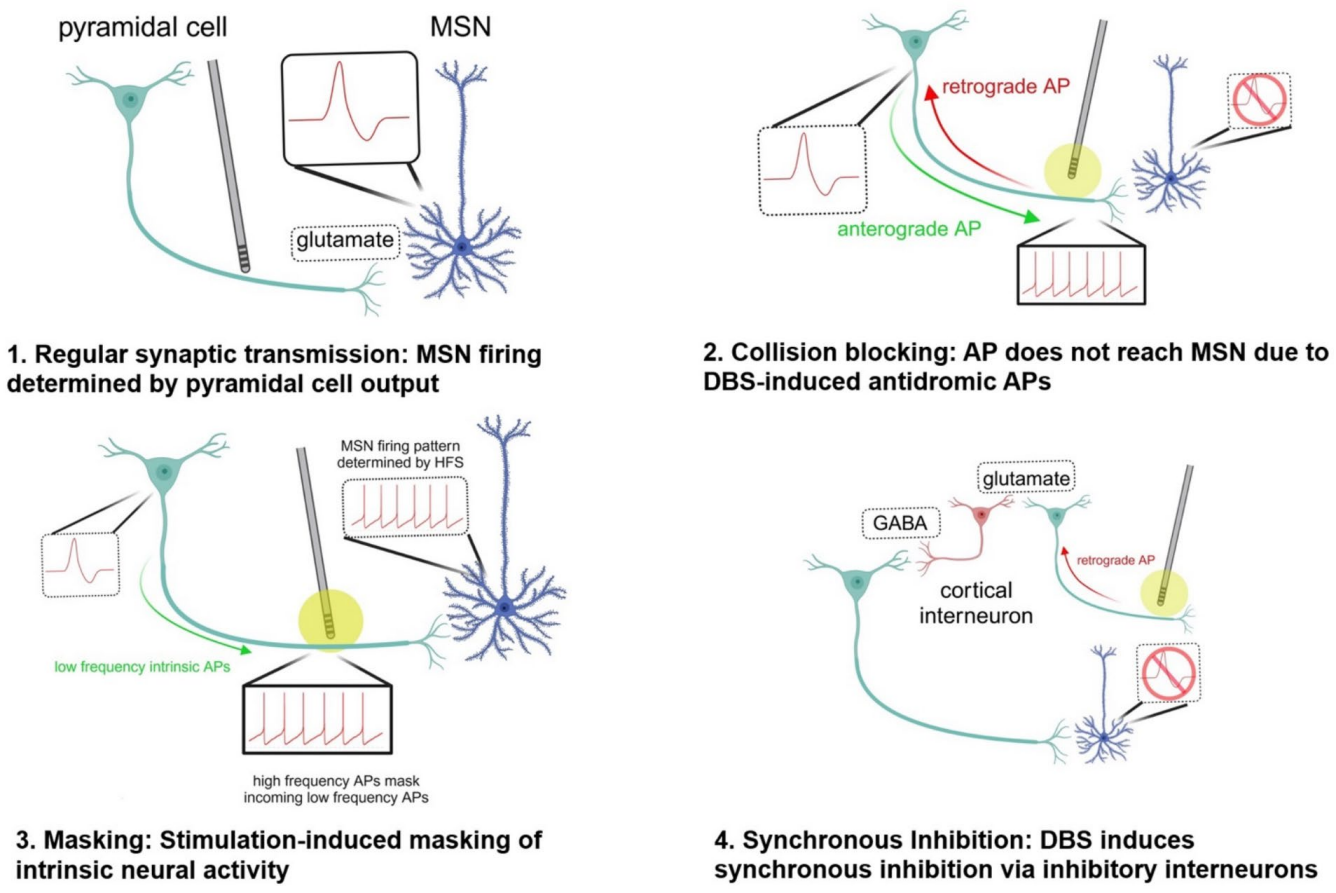
Potential mechanisms by which DBS disrupts pathological circuit activity. created in https://BioRender.com. 1. Synaptic transmission: Neural activity of MSN is determined by pyramidal cell firing in the absence of high-frequency stimulation by DBS. 2. Collision blocking: The DBS electrode induces high-frequency spiking in the axon of a pyramidal cell, preventing the propagation of anterograde action potentials down the axon. This occurs because the antidromic (retrograde) action potentials generated by DBS collide with and e!ectively block the anterograde APs that are naturally generated by the neuron. 3. Stimulation-induced masking of intrinsic neural activity: The high-frequency orthodromic action potentials traveling down the axon of the pyramidal cell stimulating the medium spiny neuron can outnumber and mask other inputs to the MSN, preventing transmission of information. 4. Synchronous inhibition: When the DBS electrode is turned on, it can generate antidromic action potentials that travel back along the axon towards the soma of the pyramidal neuron. As these antidromic signals reach branching points or synapses, they can activate local inhibitory interneurons. Synchronous inhibition of interneurons driven by high-frequency DBS may lead to increased coherent fast thalamocortical activity and reduce aberrant activity associated with OCD. AP action potential GABA gamma-aminobutyric acid HFS high-frequency stimulation MSN medium spiny neuron.

In theme, these two circuit-level mechanisms may work together or even synergistically to alleviate hyperactive CSTC circuits in OCD. Synchronized inhibition may restore intrinsic circuitry to normal firing patterns at the same time as informational lesions weaken its links (see Figure 2).

### Synthesis of DBS mechanisms in OCD

The various theories of DBS discussed – inhibition, excitation, informational lesion, and synchronized inhibition – each contribute to our understanding of how DBS affects neurons or neural circuits. While early studies supported the inhibition hypothesis, later evidence revealed that DBS can also cause excitation. The informational lesion theory reconciles these seemingly contradictory findings by proposing that DBS disrupts pathological network activity through the excitation of axons masking intrinsic activity, diminishing the importance of whether the stimulation causes local inhibition or excitation. Recent animal studies provide support for the informational lesion theory, showing that high-frequency DBS can prevent neurons from responding to intrinsic oscillations. Synchronized inhibition provides an alternative view of how circuitry might be switched to a nonpathological state. None of the theories discussed are necessarily mutually exclusive.

As mentioned previously, a hallmark and predictor of successful OCD treatment is reduced prefrontal activity. OCD patients treated with STN DBS have shown decreases in Y-BOCS scores correlating with decreases in OFC and mPFC metabolism (Bourne et al., 2012). STN DBS may modulate connections between the STN and PFC, producing these alterations in prefrontal activity. Alternatively, STN stimulation may impact subcortical structures such as the globus pallidus, a primary output structure of the basal ganglia. The final result of STN stimulation appears to be a dampening of ACC activity with therapeutic effects also related to decreased OFC metabolism. Similar mechanisms of disruption of the CSTC circuits exist for each of the stimulation sites (Bourne et al., 2012).

OCD patients have been shown to have increased oscillatory power in the slow delta and theta bands in the fronto-temporal and parietal brain regions compared with healthy controls (Perera et al., 2023). A recent study found that ALIC and NAc DBS reduced medial frontal theta activity and error-related negativity, a negative deflection seen on electroencephalogram after an incorrect response, which is also known to be altered in OCD (Perera et al., 2023; Sildatke et al., 2022).

### Linking mechanisms to clinical outcomes in OCD

Having discussed hypotheses as to the mechanisms by which DBS exerts its effects, we now turn to how the totality of these mechanisms combine to yield clinical improvement. Different stimulation targets within CSTC circuits can lead to varying clinical outcomes. VC/VS and NAc stimulation is often linked to rapid improvements in mood and anxiety, likely through direct modulation of reward pathways (Alonso et al., 2015). The VS and NAc have been shown to be hypoactive in depressive states, and DBS of the VS and NAc may modulate widespread regional brain regions connected to these structures (Sobstyl et al., 2023). In contrast, STN stimulation may have a more pronounced effect on cognitive flexibility and decision-making (Bourne et al., 2012). Anteromedial STN stimulation may regulate aberrant hyperdirect pathway activity incoming from the OFC, dlPFC, and dACC and better allow for the cortical suppression of the behaviors that are already being programmed, enabling patients to interrupt their compulsive thoughts and actions and improve cognitive flexibility and goal-directed planning. These dissociable effects underscore the importance of tailoring target selection to individual patient symptom profiles.

One study of closed-loop DBS found that VC/VS DBS also improved cognitive control, and that these improvements were correlated with a known signature of cognitive control on EEG as well as with overall clinical response to DBS (Widge, 2023). When DBS was activated, patients reported that they were more able to shift away from distressing narratives. One theoretical explanation is that VC/VS stimulation weakens CSTC circuits underlying distressing thought patterns, allowing for improved ability to maintain cognitive control. Thus, cognitive control may be improved indirectly.

### Varying time course of response to DBS

A study comparing 25 patients with severe OCD receiving DBS and 25 patients with severe OCD who declined the treatment showed an average Y-BOCS decrease of 42.5% in the treatment group and 4.8% decrease in the control group after an average follow-up period of 6.4 years (Mar-Barrutia et al., 2022). Such results demonstrate that DBS is a promising treatment for OCD, but there is an outstanding need for studies that investigate the changes in circuitry that mediate the long-term reduction in OCD symptoms over months.

The temporal pattern of DBS effects consistently shows progression from immediate to long-term improvements. ALIC stimulation for OCD shows immediate improvements in mood and anxiety, with gradual reduction in OCD symptoms over months (Greenberg et al., 2010; Tierney et al., 2014). Van Westen and colleagues further elaborated on this pattern, describing a sequence of improvements beginning with affective symptoms (seconds), followed by anxious symptoms (minutes), obsessive symptoms (days), and finally compulsions (weeks or months) (van Westen et al., 2015).

The variable time course for different symptoms suggests that there are multiple mechanisms at play (Herrington et al., 2016). Symptoms that respond immediately are likely mediated by neuromodulation of pathological network activity (Herrington et al., 2016), while longer term results are likely mediated by synaptic plasticity and anatomic remodeling (Agnesi et al., 2013; Temperli et al., 2003). Rapid mood enhancement observed with NAc DBS can be attributed to immediate modulation of limbic circuits, particularly those connecting the VC/VS or NAc with the prefrontal cortex (Fridgeirsson et al., 2020). At high voltages, this modulation can lead to impulsivity and manic behaviors such as excessive spending (Figee et al., 2014; Luigjes et al., 2011). Mood and anxiety improvements are associated with decreased amygdala-insula connectivity and increased communication between the ventromedial prefrontal cortex and the amygdala (Fridgeirsson et al., 2020). In contrast, OCD symptom improvement may involve more gradual changes across multiple domains, including striatal dopaminergic and habenular serotonergic circuits (Zhang et al., 2020).

A recent review article notes that low-frequency oscillations are reinforced through long-term potentiation, whereas high-frequency stimulation has a lesser effect on plasticity (Tass and Majtanik, 2006). Thus, replacement of low-frequency activity with high-frequency activity might allow for the rewriting of circuitry. However, unlike motor disorders, there is little evidence to support an association between psychiatric diseases and low-frequency activity within basal ganglia–cortical circuits, which leaves open the possibility that DBS might also work through other mechanisms (Lozano et al., 2019).

One area of current interest is the effects of DBS on astrocytes, given their role in integrating synaptic information and regulating synaptic plasticity (Ashkan et al., 2017). Changes induced by astrocytes could help explain the delayed and progressive nature of the benefits of DBS treatment. Interest is growing in the neuroplastic changes induced by DBS that could upregulate the expression of trophic and synaptic proteins (Gondard et al., 2015).

## Conclusion and future directions

This review has explored the mechanisms underlying the efficacy of DBS in treating OCD. We have traced the evolution of theories from initial hypotheses of local excitation and inhibition to more nuanced ideas of circuit modulation. The pathophysiology of OCD involving CSTC circuits provides a framework for understanding why circuit modulation is a promising avenue for treatment. Recent animal studies have illuminated how DBS may create informational lesions that disrupt intrinsic oscillations. While significant progress has been made in understanding DBS mechanisms, many questions remain. It is unclear how different potential mechanisms of circuit modulation such as informational lesions and synchronized inhibition work together. In addition, it is unknown how the short-term effects on circuits observed in animal models relate to long-term clinical improvement in OCD patients. Is the pattern of short-term changes and circuit modulation in animal studies homologous to a more permanent engraving in the brain of patients with OCD receiving DBS, or does long-term stimulation have its own signature of synaptic plasticity and subsequent anatomic change that have not been studied? Future research should focus on bridging the gap between short-term and long-term neurobiological signatures, as well as exploring how these signatures correlate to the time course of clinical improvement on axes such as mood and anxiety versus OCD-specific symptomatology. In addition, more research is needed in newer areas of inquiry such as the effects of DBS on astrocytes. Finally, although parameters can be derived from assumptions about the target volume and target elements, modeling the interactions between stimulation of varying parameters and the disordered neural communication that underlies OCD is much more difficult to develop. To this end, it has been suggested that a registry be developed to gather more precise data about patient clinical profiles, stimulation site, parameters, and outcomes (Synofzik et al., 2012).

Evolving understanding of DBS mechanisms may lead to optimized stimulation targets, parameters, and patient selection for this relatively novel psychiatric treatment. Ultimately, a deeper understanding of DBS mechanisms may not only improve outcomes for patients with OCD but also contribute to our broader knowledge of brain function and neuropsychiatric disorders.
